# Clinical Efficacy of Acupuncture for the Treatment of Rheumatoid Arthritis: Meta-Analysis of Randomized Clinical Trials

**DOI:** 10.1155/2022/5264977

**Published:** 2022-04-30

**Authors:** Hongchao Li, Siliang Man, Liang Zhang, Lidong Hu, Hui Song

**Affiliations:** ^1^Department of Rheumatology, Beijing Jishuitan Hospital, Beijing 100035, China; ^2^Department of Orthopedics, Beijing Jishuitan Hospital, Beijing 100035, China; ^3^School of Medicine, Xiamen University, Xiamen 361102, China

## Abstract

**Objective:**

Acupuncture has been used by rehabilitation specialists as an adjunctive treatment for the symptomatic treatment of rheumatoid arthritis (RA). This meta-analysis aims to evaluate the efficacy of acupuncture in treating patients with RA.

**Methods:**

A comprehensive search was conducted in CBM, CNKI, PubMed, CENTRAL, Web of Science, and Embase from their inception up to March 2022. All randomized controlled trials (RCTs) without the language restriction, concerning the patients with RA treated with acupuncture, were included. Two reviewers independently assessed the risk of bias with the Cochrane Risk of Bias Assessment tool. Weight mean difference (MD) and 95% CI were calculated, and data were pooled with random effects model.

**Results:**

A total of eleven RCTs involving 796 patients with RA met the established inclusion criteria. This systematic review indicated the efficacy of acupuncture as an adjunctive treatment for patients with RA. Invasive acupuncture could reduce significantly in pain (MD = -1.00, 95% CI: −1.96 to −0.05, *P*=0.040), health assessment questionnaire (HAQ, MD = −0.20, 95% CI: −0.30 to −0.11, *P* < 0.001), physician global assessment (PhGA, MD = −0.98, 95% CI: −1.23 to −0.72, *P* < 0.001), tender joint count (TJC, MD = −1.24, 95% CI: −2.11 to −0.37, *P*=0.005), C-reactive protein (CRP, MD = −1.81, 95% CI: −3.32 to −0.29, *P*=0.019), and erythrocyte sedimentation rate (ESR, MD = −3.03, 95% CI: −5.80 to −0.26, *P*=0.032), while compared to control group. Laser acupuncture could reduce HAQ (MD = −0.15, 95% CI: −0.28 to −0.01, *P*=0.034), the RA quality of life questionnaire (RAQoL, MD = −2.32, 95% CI: −4.40 to −0.25, *P*=0.028), CRP (MD = −35.24, 95% CI: −36.49 to −33.99, *P* < 0.001), and interleukin-6 (IL-6, MD = −29.63, 95% CI: −49.34 to −9.92, *P*=0.003), while compared to control group. No adverse events associated with acupuncture were reported.

**Conclusion:**

Available evidence suggests that acupuncture is beneficial for relieving pain and ameliorating quality of life and health index in patients with RA; thereby, it should be available as an adjunctive nonpharmacological treatment in rehabilitation programmes.

## 1. Introduction

Rheumatoid arthritis (RA), as a chronic inflammatory autoimmune disease, is manifested by pain, stiffness, swelling, and impaired joint function in the peripheral joints [1]. This chronic painful disease not only seriously damages patients' quality of life and mental conditions but is also one of the leading causes of joint deformity or disability [2]. Currently, RA has been a major public health issue affecting about 1% of the world's population [3]. In clinical practice, RA is typically treated with NSAIDs, csDMARDs, glucocorticoids, and biologics. However, when longtime medications are given for treatment, patients are not free from adverse effects and exhibit a high degree of variability in therapeutic responses such as malignancy, immunosuppression, liver damage, bone marrow dysfunction, life-threatening infections, hyperglycemia, interstitial lung disease, and hypertension. Therefore, patients often go in quest of more nonpharmacological treatments as complementary and alternative therapy. As a mainstay of the nonpharmacological treatment approach, acupuncture has since gained ever-growing attention in the treatment of RA.

Acupuncture is a unique treatment method based on the practice of traditional Chinese medicine, in which needles are inserted into certain specific locations of the exterior body, namely acupoints, to relieve pain or for other therapeutic purposes. Based on the theory of traditional Chinese medicine, RA is diagnosed as “*Bi*” syndrome caused by the invasion of wind, cold, dampness, or heat pathogen into the body's meridians, and then affects joints, bones, and muscles, manifested as articular pain, swollen, stiffness, and even deformity. Acupuncture has been used to treat “*Bi*” syndrome for thousands of years based on traditional Chinese medicine theory, but its mechanism of action has not yet been elucidated. Current study indicated that acupuncture provided nonanalgesic effect mainly via suppressing the inflammatory response, improving blood flow, and relaxing muscle tone [4], which needs to be further confirmed. With the development of technology, current acupuncture includes not only traditional acupuncture but also electroacupuncture, intensive acupuncture, laser acupuncture, etc.

There are relatively few systematic reviews and meta-analyses supporting the application of acupuncture to the symptomatic treatment of the patients with RA, and these evidences have methodologic concerns and suspicious conclusions about the effectiveness of acupuncture on the treatment of RA. Besides, these studies were mainly systematic review of randomized controlled trials (RCTs) without meta-analysis, and most recent systematic review on clinical efficacy of acupuncture for the treatment of rheumatoid arthritis is about five years ago. Hence, this meta-analysis aims to summarize the latest state of scientific evidence based on published RCTs worldwide and potentially find scientific proof that will allow us to conclude whether acupuncture is effective in relieving pain and improving the quality of life in patients with RA.

## 2. Materials and Methods

We followed strictly the recommendations of the Cochrane Collaboration and the PRISMA (Preferred Reported Items for Systematic Reviews and Meta-Analyses) guidelines to perform this meta-analysis. A comprehensive literature review was conducted to identify all data on the clinical efficacy of acupuncture treatment in patients with RA from published RCTs.

### 2.1. Systematic Literature Retrieval

Systematic literature search was performed in Chinese Biomedical Database (CBM), CNKI, PubMed, CENTRAL, Web of Science, and Embase to identify available studies up to March 2022. The search strategy focused on rheumatoid arthritis and acupuncture and was limited to humans and RCTs published in Chinese and English. All bibliographic references cited in RCTs and meta-analysis of interest were tracked to identify additional citations that were not identified through the above search strategy.

### 2.2. Eligibility Criteria

#### 2.2.1. Type of Study Design

RCTs using acupuncture as the treatment of RA.

#### 2.2.2. Type of Patients

Patients (more than 18 years old) with RA diagnosed by physician based on the diagnostic criteria of the European League Against Rheumatism and American College of Rheumatology.

#### 2.2.3. Type of Intervention

Studies intervened with needle acupuncture with or without electrical stimulation or moxibustion, or laser at precise locations to stimulate acupoints for the purpose of treatment. The control group received the same interventions as the acupuncture group, except for acupuncture, or penetrating sham acupuncture.

#### 2.2.4. Outcomes

The primary outcomes included C-reactive protein (CRP), health assessment questionnaire (HAQ), visual analogue scale (VAS) of pain, and the RA quality of life questionnaire (RAQoL). If possible, secondary outcomes, such as swollen joint count (SJC), tender joint count (TJC), physician global assessment (PhGA), erythrocyte sedimentation rate (ESR), and interleukin-6 (IL-6), were also measured and assessed.

### 2.3. Study Selection and Data Extraction

Two reviewers (H.L. and S.M.) independently extracted the data of all eligible studies, and other two reviewers (L.Z. and L.H.) checked the extracted results. Any discrepancy was settled through discussion and consensus, with the third reviewer acted as an adjudicator (H.S.), if necessary. Furthermore, we collected general information on each available study such as authors' name, study design, country, year of publication, sample size, age and sex distribution of patients, and intervention characteristics (such as details and duration of interventions and controls and follow-up) and their outcomes.

### 2.4. Assessment of the Risk of Bias

The risk of bias for all eligible studies was independently evaluated by two reviewers (H.L. and S.M.) according to the Cochrane Risk of Bias Assessment tool, version 2. It was mainly evaluated through the following domains of each study: randomization, allocation concealment, blinding, incomplete outcome data, selective reporting, other biases such as baseline imbalance, and funding. We evaluated the risk of bias for each available study as low risk of bias, high risk of bias, and unclear risk of bias due to uncertainty on the potential bias or lack of information.

### 2.5. Statistical Analysis

This meta-analysis was conducted based on at least two studies with *R* software version 4.0.2. Weighted mean difference (WMD) and its 95% confidence interval (CI) were calculated for the mean changes from the baseline of outcomes in the acupuncture group and control group. The Cochran *Q* statistic test was used to assess the heterogeneity across RCTs. The heterogeneity was quantified with the *I*^2^ index. If *I*^2^ value was over 50%, substantial heterogeneity was considered to exist [5]. According to the recommendations of Borenstein et al. [6] and considering the actual situation of the included studies, random effects model was used to pool data. Two-tailed *P* < 0.05 was interpreted as significant.

## 3. Results

### 3.1. Literature Selection and Trial Characteristics

**Figure 1:**
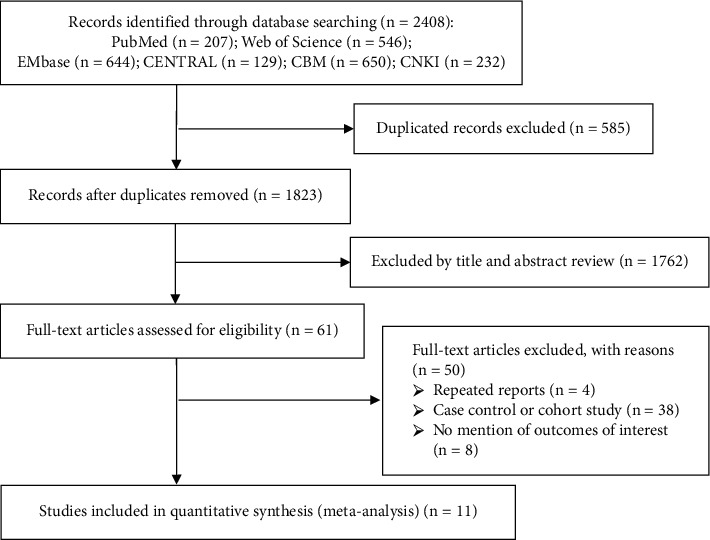
Flow chart of literature selection.

We identified initially 2408 unique citations located through these databases. In our first selection round, 1823 records were potentially related to our topic, with 61 being considered eligible for a full-text review. Eleven RCTs ultimately met the inclusion criteria for this meta-analysis [7–17]. All the selected RCTs were reported in English and Chinese. The PRISMA flow diagram for literature screening is shown in Figure 1.

All the included studies were published between 2006 and 2021. Of 796 patients in the eligible eleven studies, 402 patients received acupuncture (including electroacupuncture, traditional acupuncture, and laser acupuncture), and 394 patients received control interventions (including sham acupuncture, medicine, exercise, and others). The average age of the included studies ranged between 46.5 and 69.1 years. The main characteristics of all the selected studies are summarized in Table 1.

### 3.2. Risk of Bias Assessments

**Figure 2:**
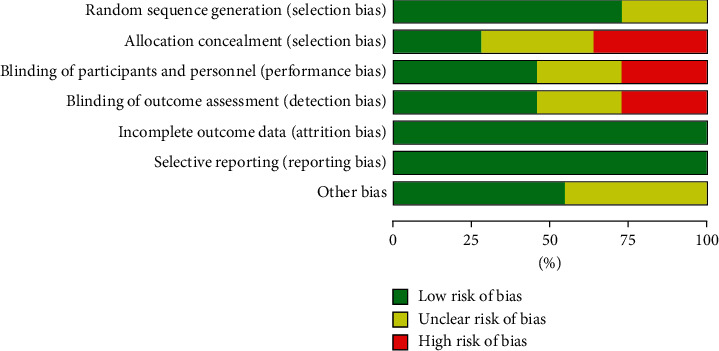
Risk of bias graph.

The results of the risk of bias assessment of the eight studies are illustrated in Figure 2. Three trials simply mentioned randomization methods used in their research and omitted details concerning random sequence generation methods [11, 12, 17]. Only three trials indicated what allocation concealment methods were used to maintain privacy after random allocation [7, 9, 10]. Five trials reported the blinding of personnel and outcome assessment [7–10, 13]. All studies had a low risk of bias in incomplete outcome data and selective reporting.

### 3.3. Outcomes Measures

#### 3.3.1. Pain

**Figure 3:**
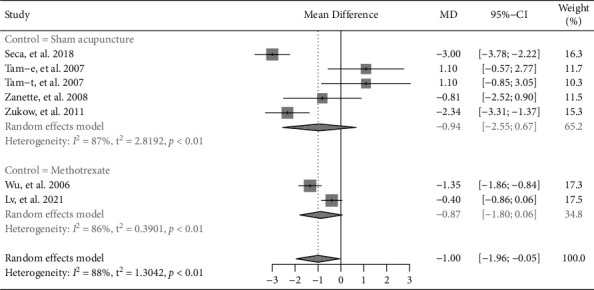
Forest plot of meta-analyses comparing invasive acupuncture versus control on pain reduction.

Six trials reported pain experienced by patients using VAS [8–10, 13]. The pooled results of these trials showed that the pain score was significantly reduced in patients with RA who were treated with invasive acupuncture, while compared to control group (MD = −1.00, 95% CI: −1.96 to −0.05,*P*=0.040) (Figure 3). However, subgroup analysis based on invasive or noninvasive intervention in control group showed that there was no significant difference between invasive acupuncture group and control group (invasive acupuncture vs. sham acupuncture: MD = −0.94, 95% CI: −2.55 to 0.67, *P*=0.25; invasive acupuncture vs. methotrexate: MD = −0.87, 95% CI: −1.80 to 0.06, *P*=0.067).

#### 3.3.2. Quality of Life

**Figure 4:**
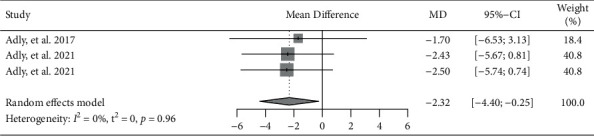
Forest plot of meta-analyses comparing laser acupuncture versus control on improvement of quality of life (RAQoL).

Three trials reported the quality of life of the patients with RA measured by RAQoL [7, 11, 12]. The results of meta-analysis indicated that laser acupuncture had significantly better effects in ameliorating the quality of life, while compared to the control group (MD = −2.32, 95% CI: −4.40 to −0.25, *P*=0.028) (Figure 4).

#### 3.3.3. Health Status

**Figure 5:**
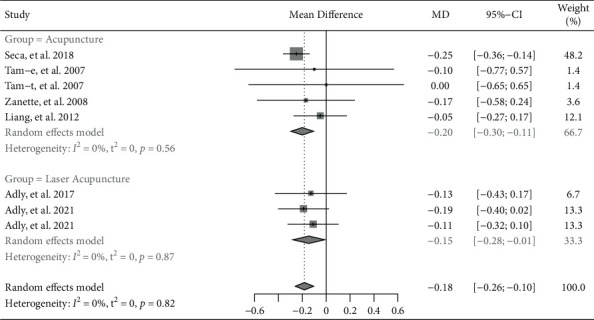
Forest plot of meta-analyses comparing acupuncture versus control on improvement of health status (HAQ).

Seven trials reported the health assessment of patients with RA measured by HAQ [7–12, 17]. The pooled result indicated that acupuncture had significantly better effects in ameliorating health status, while compared to the control group (MD = -0.18, 95% CI: −0.26 to −0.10, *P* < 0.001) (Figure 5). Subgroup analysis based on invasive or noninvasive intervention in the experimental group showed that invasive acupuncture had significantly better effects in ameliorating health status (MD = −0.20, 95% CI: −0.30 to −0.11, *P* < 0.001), and laser acupuncture had also significantly better effects in ameliorating health status (MD = −0.15, 95% CI: −0.28 to −0.01, *P*=0.034). Besides, subgroup analysis based on invasive or noninvasive intervention in the control group showed that invasive acupuncture had significantly better effects in ameliorating health status (invasive acupuncture vs. sham acupuncture: MD = −0.23, 95% CI: −0.34 to −0.13, *P* < 0.001; except for invasive acupuncture vs. oral medication: MD = −0.05, 95% CI: −0.27 to 0.17, *P*=0.661) (Supplementary Figure 1).

#### 3.3.4. Joint Involvements

**Figure 6:**
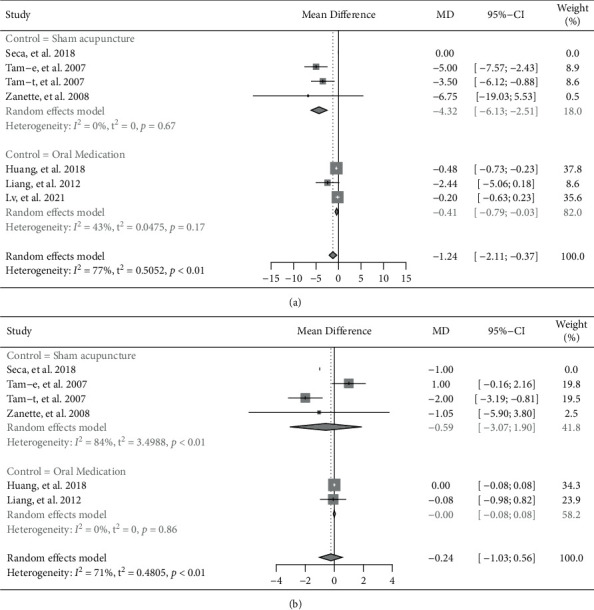
Forest plot of meta-analyses comparing invasive acupuncture versus control on reduction of joint involvements. (a) Tender joint count; (b) swollen joint count.

Seven trials reported tender joint count (TJC) [8–10, 14, 16, 17]. The pooled results of these trials showed a significant reduction on TJC in patients with RA who were treated with acupuncture, while compared to the control group (MD = −1.24, 95% CI: −2.11 to −0.37, *P*=0.005) (Figure 6(a)). Subgroup analysis based on invasive or noninvasive intervention in the control group showed that invasive acupuncture had significantly better effects in reducing TJC (invasive acupuncture vs. sham acupuncture: MD = −4.32, 95% CI: −6.13 to −2.51, *P* < 0.001; invasive acupuncture vs. oral medication: MD = −0.41, 95% CI: −0.79 to −0.03, *P*=0.035).

Six trials reported swollen joint count (SJC) [8–10,14,17]. The pooled results of meta-analysis suggested no significant difference between the invasive acupuncture group and the control group (MD = −0.24, 95% CI: −1.03 to 0.56, *P*=0.580) (Figure 6(b)). Besides, subgroup analysis based on invasive or noninvasive intervention in the control group showed also no significant difference between the invasive acupuncture group and the control group (invasive acupuncture vs. sham acupuncture: MD = −0.59, 95% CI: −3.07 to 1.90, *P*=0.642; invasive acupuncture vs. oral medication: MD = −0.00, 95% CI: −0.08 to 0.08, *P*=0.988).

#### 3.3.5. Inflammatory Markers

**Figure 7:**
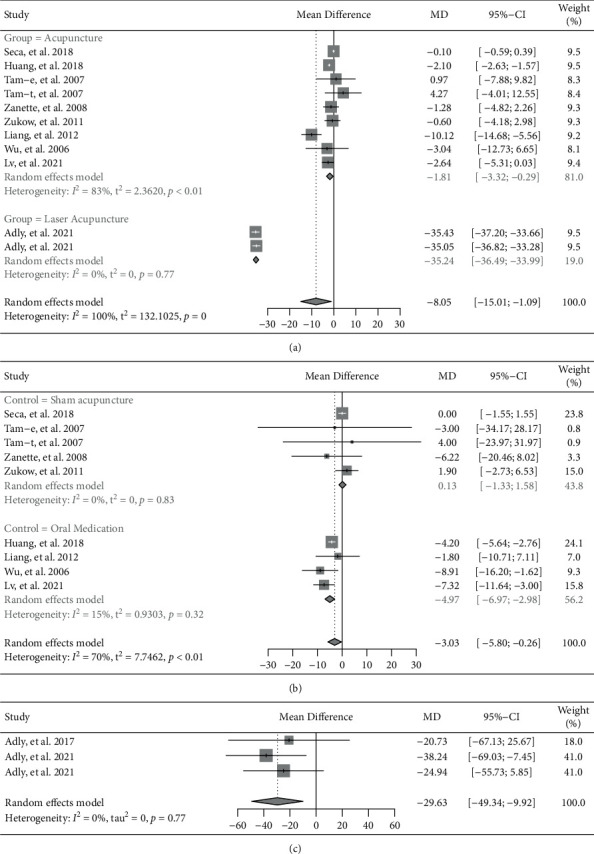
Forest plot of meta-analyses comparing acupuncture versus control on reduction of Inflammatory markers. (a) CRP; (b) ESR; (c) IL-6.

Eleven trials reported CRP [8–10,13–17]. The pooled results in this meta-analysis indicated a significant reduction in the acupuncture group, while compared to the control group (MD = −8.05, 95% CI: −15.01 to −1.09, *P*=0.023) (Figure 7(a)). Subgroup analysis based on invasive or noninvasive intervention in the experimental group showed that invasive acupuncture had significantly better effects in reducing CRP (MD = −1.81, 95% CI: −3.32 to −0.29, *P*=0.019), and laser acupuncture had also significantly better effects in reducing CRP (MD = −35.24, 95% CI: −36.49 to −33.99, *P* < 0.001).

Nine trials reported ESR [8–10,13–17]. The pooled results indicated a reduction on ESR in invasive acupuncture group (MD = −3.03, 95% CI: −5.80 to −0.26, *P*=0.032) (Figure 7(b)). Subgroup analysis based on invasive or noninvasive intervention in control group showed that invasive acupuncture had significantly better effects in reducing ESR (invasive acupuncture vs. oral medication: MD = −4.97, 95% CI: −6.97 to −2.98, *P* < 0.001; except for invasive acupuncture vs. Sham acupuncture: MD = 0.13, 95% CI: −1.33 to 1.58, *P*=0.864).

Three trials reported serum IL-6 [7,11,12]. The pooled result indicated that laser acupuncture had significantly better effects in decreasing serum IL-6, while compared to the control group (MD = −29.63, 95% CI: −49.34 to −9.92, *P*=0.003) (Figure 7(c)).

#### 3.3.6. PhGA

**Figure 8:**
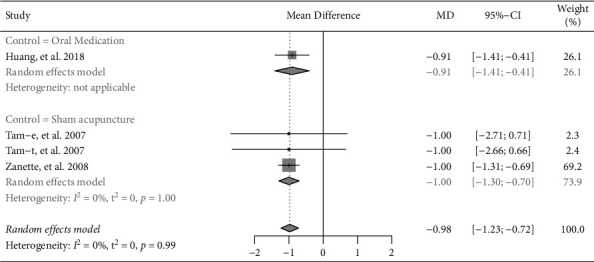
Forest plot of meta-analyses comparing acupuncture versus control on physician global assessment (PhGA).

Four trials reported PhGA [8,9,14]. The pooled result indicated that PhGA score was significantly reduced for the invasive acupuncture group, while compared to the control group (MD = −0.98, 95% CI: −1.23 to −0.72, *P* < 0.001) (Figure 8). Subgroup analysis based on invasive or noninvasive intervention in the control group showed that invasive acupuncture had significantly better effects in reducing PhGA (invasive acupuncture vs. oral medication: MD = −0.91, 95% CI: −1.41 to −0.41, *P* < 0.001; invasive acupuncture vs. sham acupuncture: MD = −1.00, 95% CI: −1.30 to −0.70, *P* < 0.001).

#### 3.3.7. Adverse Effects

No adverse effects associated with acupuncture were reported.

## 4. Discussion

RA, as an autoimmune musculoskeletal disease, mainly manifests as symmetrical persistent synovitis, rheumatic pain, and destructive polyarthritis involving synovium [18,19]. Without active treatment, RA may lead to joint destruction, limited physical function, and even severe disability [19]. However, no cure is currently available for patients with RA, and treatment options include both pharmacological and nonpharmacological treatments. Pharmacological agents for alleviating symptoms may lead to serious adverse events (e.g., myelosuppression, liver and kidney impairment, gastrointestinal bleeding, and infections) and may impose a heavy economic burden on patients, which may limit the compliance. Consequently, patients with RA still need the alternative and complementary treatments to gain additional relief and the treatments with fewer side effects. Approximately 60% to 90% of patients with arthritis are being treated with some forms of alternative and complementary medicine, including acupuncture [20]. Acupuncture is a widely used nonpharmacological therapy in China due to the advantages of safety, reliability, and easy operation.

According to the theory of traditional Chinese medicine, RA patients should meet the conditions of so-called “*Bi*” syndrome, that is, it means that blockage of the meridians caused by any disease pattern to occur due to wind cold dampness evil invading the fleshy exterior and joints, manifesting as some specific symptoms like sinew pain, joint pain, and bone pain, as well as numbness or heaviness of patient's limbs [21]. Acupuncture has been shown to be effective to remove “blood” (*xue*) stasis for arthritis patients such as osteoarthritis. Acupuncture is defined as stimulating a certain point or certain points on the body, known as acupoints, through the insertion and twisting of the needles to achieve desirable therapeutic effects. This is believed to be able to change or prevent the perception of pain or to modify psychophysiological functions to some extent, including pain and mental controls for the treatments of certain diseases or physical dysfunction. Some authors have already used different acupuncture manipulations, such as moxibustion, needle-sticking method, warm needling, plus herb steaming, and reinforcing-reducing/twirling-reinforcing needling to treat certain chronic painful diseases, and achieved better clinical efficacy [22–24]. In addition, special forms of acupoints stimulation include laser, electroacupuncture, millimeter wave, and ginger-salt-partitioned moxibution [12,25–27].

Acupuncture seems to be a promising treatment while compared to conventional current RA medication. Our meta-analysis indicated that the selected acupoints were obviously different among these clinical studies, and the most commonly used acupoints were ST36 (63.6%) and LI11 (45.5%). Most studies showed that acupuncture could improve pain, although some studies did not indicate statistical significance, except for the study by Tam et al. Besides, this meta-analysis also pointed out that the use of acupuncture might ameliorate the HAQ, RAQoL, PhGA, tender joint count, and inflammatory markers of patients with RA, while compared to control management strategies. Traditional Chinese Medicine treatment follows the people-centered principle, so the treatment protocol of acupuncture is not fixed, and it is always adjusted according to the different Traditional Chinese Medicine syndrome patterns that corresponded to different biological processes. In the treatment protocol of Zukow et al., 20 acupoints were selected and the subsequent treatment was adjusted according to the patient's symptoms, and the results showed that acupuncture was superior to sham acupuncture in pain improvement. The study by David et al. selected only one acupoint (LI3) to observe the therapeutic effects of acupuncture and concluded that acupuncture was ineffective in relieving patients' pain. They themselves believed that the acupoint LI3 may be an incorrect or inadequate point on its own to use in order to show an effect in RA. This meta-analysis results showed that there was heterogeneity among many research results, which may be related to the selection of acupoints, operation technology, course of treatment, and acupuncture combined with other therapies.

In patients with RA, the core mechanism of chronic nociceptive pain is thought to be inflammation. In patients with RA, inflammatory mediators generally tend to accumulate in the synovial fluid of the joints, and then, these inflammatory mediators can directly activate the nerve terminals within these joints, leading to the release of neurotransmitters and the transmission of pain messages and consequently to inflammatory pain. Previous studies have indicated that the acupuncture at acupoints can induce certain cells proliferation and migration, especially for fibroblasts, and modulate the production and secretion of cytokines, growth factors, and inflammatory mediators such as CRP, TNF-*α*, and IL-6. Besides, it can also decrease oxidative stress, improve tissue oxygenation, and relieve pain [28,29]. It has been suggested that IL-6 played a vital role in the development and progression of RA and was especially responsible for the progressive destruction of articular cartilage and bone [30]. Acupuncture can effectively reduce the level of IL-6 in peripheral blood of the patients with RA, which demonstrates that it has a high anti-inflammatory activity in the treatment of RA, which was in line with our results [30,31]. Besides, a study reported the use of buccal acupuncture in RA rabbits and found that cholecystokinin-8 and endorphin in cerebrospinal fluid were upregulated with needles retaining for 30 minutes, and they had a central analgesic effect [32]. This is a very short-term observation, but the effect is very good. Another study indicated that acupuncture could reduce the levels of VEGF and TNF-*α* in joint synovia and peripheral blood and ameliorate the internal environment of the body, which is very benefit to patients with RA [29].

In addition, studies suggested that acupuncture may work by stimulating the serotonergic, noradrenergic, and opioid system and promoting the release of endorphins, which may help relieve pain and increase feelings of well-being and relaxation in this way [33,34]. It is well known that one of the symptoms of patients with RA is the progressive development of various degrees of anxiety and depression. The results in a rat model of chronic painful cold stress indicated that acupuncture at stomach meridian point 36 could directly affect the hypothalamus-pituitary-adrenal axis without altering feedback systems and thus effectively inhibit the release of stress hormones, which could reduce depression- and anxiety-related behaviors [35]. Another study suggested that acupuncture as a therapeutic approach might enhance and harmonize the body, mind, and spirit, further significantly improving the health and well-being of patients, and had favorable effects on the outcomes, prognosis, and rehabilitation of diseases [36].

There are several limitations to consider. First, as shown in Figure 2, many trials included did not report in detail or used appropriately allocation concealment and blinding, which may affect the testing power of research results. Second, due to the major inherent limitation in these included short-term exposure RCTs, these findings represent only a short- or midterm evaluation of the clinical efficacy of acupuncture treatment in the patients with RA. Third, there are few relevant studies, and the quality of methodology is often not high, which will greatly limit the conclusiveness of this study.

In summary, this meta-analysis shows that acupuncture is beneficial for relieving pain and ameliorating the quality of life and health index in patients with RA; thereby, it should be available as an adjunctive nonpharmacological treatment in rehabilitation programmes. In addition, due to limitations of paucity and suboptimal methodology of the primary studies, our findings need to be verified by some studies with larger cohorts and longer follow-up periods.

## Figures and Tables

**Table 1:** Characteristics of randomized controlled trials included in the systematic review

Study	Year	Country	Sample	Age, mean ± SD		Female, n(%)		Follow-up	ClinicalTrials.gov	Acupoints	Intervention	Control
				Acupuncture	Control	Acupuncture	Control					
Adly et al.	2021	Egypt	26/34	68.8 ± 2.6	69.1 ± 2.8	18 (60.0)	23 (76.6)	4 weeks	NCT04684693	ST25, LR8, SP6, SP9, and PC6	Laser acupuncture + aerobic exercise	Aerobic exercise
Zanette et al.	2008	Brazil	20/20	53.1 ± 12.4	46.5 ± 9.9	17 (85)	20 (100)	9 weeks	/	Supine position: EX1, PC6, IG4, EX28, CV12, CV6, ST36, SP6, and LV3; Ventral position: UB20, UB22, UB23, GV4, GV14, UB11, and UB60	Acupuncture (20 min, 2 times weekly for 5 weeks) plus analgesia	Penetrating sham acupuncture (nonacupoints, minimal, 20 min, 10 times) plus analgesia
Tam et al.	2007	China	12/12/12	56.4 ± 8.5 58.1 ± 12.0	57.6 ± 8.3	11 (91.7) 10 (83.3)	8 (66.7)	10 weeks	NCT00404443	LI11, TE5, LI4, ST36, GB34, and GB39	Acupuncture (40 min, 2 times weekly for 10 weeks) plus analgesia	Penetrating sham electroacupuncture plus analgesia
Seca et al.	2019	Portugal	35/35	57.3 ± 11.3	56.5 ± 16.4	31 (88.6)	30 (85.7)	4 weeks	NCT02553005	TB5, GB39, HT3, and KI7	Verum acupuncture (2 times weekly for 4 weeks)	Penetrating sham acupuncture (2 times weekly for 4 weeks)
Adly et al.	2021	Egypt	30/30	68.9 ± 2.7	69.1 ± 2.9	41 (68.3)	4 weeks	NCT04758689	ST36, LR8, SP9, and PC6	Laser acupuncture + aerobic exercise + methotrexate	Aerobic exercise + methotrexate	
Adly et al.	2017	Egypt	15/15	61.9 ± 2.6	63.2 ± 3.7	22 (73.3)	4 weeks	/	LR3, ST25, ST36, SI3, SI4, LI4, LI11, SP6, SP9, GB25, GB34, and HT7	Laser acupuncture (12 sessions over 4 weeks)	Reflexology therapy (12 sessions over 4 weeks)	
Zukow et al.	2011	Poland	35/32	58.1 ± 11.5	57.6 ± 9.2	33 (94.3)	32 (91.4)	22 weeks	/	LI3, LI4, LI10, LI11, TE5, ST36, SP 4, SP6, BL7, BL43, BL56, BL60, BL62, GB34, GB38, GB39, GB41, GV4, GV14, and GV20	Acupuncture (30-45 min)	Penetrating sham acupuncture (30-45 min)
Huang et al.	2018	China	81/82	48 ± 5	49 ± 8	64 (79.0)	63 (76.8)	4 weeks	/	/	Acupuncture + Zhuang medicine (7 times weekly for 4 weeks) + csDMARDs	csDMARDs
Wu et al.	2006	China	45/45	52 ± 12	51 ± 11	36 (80.0)	35 (77.8)	5 months	/	SJ14, SI9, LI15, LU5, LI11, PC3, SJ4, SI5, LI5, EX-UE9, EX-LE5, LR8, GB33, ST36, GB34, KI3, ST41, BL60, GB40, EX-LE10, and ST7	Acupuncture (once every other day for 30 m in)	Methotrexate
Lv et al.	2021	China	51/49	41 ± 14	41 ± 14	33 (64.7)	24 (71.4)	12 weeks	/	RN12, RN10, RN4, RN6, ST24, and ST26	Bo's abdominal acupuncture (every other day) + Methotrexate	Methotrexate
Liang et al.	2012	China	40/40	40 ± 9	38 ± 9	31 (77.5)	32 (80.0)	4 weeks	/	BL20, BL23, ST36, CV4, Ex-LE10, LI5, PC7, LI11, TE10, LI15, TE14, Ex-LE5, ST41, and Ex-UE9	Acupuncture (30 min, 5 times weekly for 4 weeks) + Meloxicam + Methotrexate + Leflunomide	Meloxicam + Methotrexate + Leflunomide

## Data Availability

The data used to support the findings of this study are available from the corresponding author upon request.

## Supplementary Materials

Supplementary Materials Supplementary Figure 1: forest plot of meta-analyses comparing acupuncture versus control on improvement of health status (HAQ) based on invasive or noninvasive intervention in the control group.
