# Supplementary figures and images for: Clinical Efficacy of Acupuncture for the Treatment of Rheumatoid Arthritis: Meta-Analysis of Randomized Clinical Trials
Source: Evid Based Complement Alternat Med. 2022 Apr 30;2022:5264977. doi: 10.1155/2022/5264977 (PMC9078778; doi:10.1155/2022/5264977)

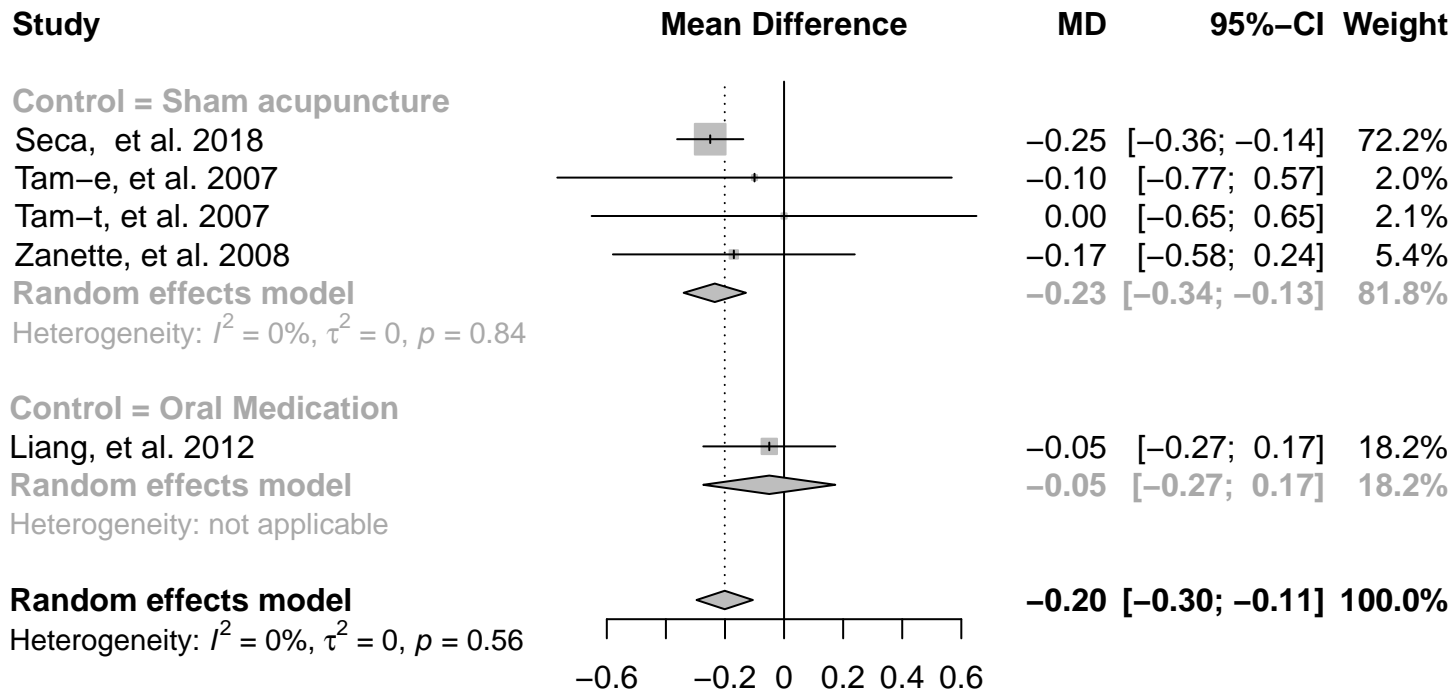

Supplement: Supplementary Materials — Supplementary Figure 1: forest plot of meta-analyses comparing acupuncture versus control on improvement of health status (HAQ) based on invasive or noninvasive intervention in the control group. [file 5264977.f1.pdf]
